# HIV’s Nef Interacts with β-Catenin of the Wnt Signaling Pathway in HEK293 Cells

**DOI:** 10.1371/journal.pone.0077865

**Published:** 2013-10-10

**Authors:** Keren Weiser, Meredith Barton, Dafna Gershoony, Ramanuj DasGupta, Timothy Cardozo

**Affiliations:** Department of Biochemistry and Molecular Pharmacology, New York University School of Medicine, New York, New York, United States of America; University of Texas Medical Branch, United States of America

## Abstract

The Wnt signaling pathway is implicated in major physiologic cellular functions, such as proliferation, migration, cell fate specification, maintenance of pluripotency and induction of tumorigenicity. Proliferation and migration are important responses of T-cells, which are major cellular targets of HIV infection. Using an informatics screen, we identified a previously unsuspected interaction between HIV’s Nef protein and β-catenin, a key component of the Wnt pathway. A segment in Nef contains identical amino acids at key positions and structurally mimics the β-catenin binding sites on endogenous β-catenin ligands. The interaction between Nef and β-catenin was confirmed *in vitro* and in a co-immunoprecipitation from HEK293 cells. Moreover, the introduction of Nef into HEK293 cells specifically inhibited a Wnt pathway reporter.

## Introduction

Nef is an accessory protein encoded by the human (HIV-1 and HIV-2) and simian immunodeficiency (SIV) viruses and is an essential mediator of viral pathogenicity [1,2]. This protein helps maintain high viral loads and overcome host immune defenses, thereby contributing to the progression of AIDS [2,3]. Patients infected with *nef*-deleted HIV-1 strains develop AIDS symptoms much more slowly than those infected with standard HIV strains [4,5], and experimental deletion within the SIV *nef* gene reduces viral load, delays the onset of an AIDS-like disease, and offers immune protection against infection with pathogenic SIV in the rhesus macaque animal model of HIV [6]. The molecular basis for these effects is unknown.

Nef modulates the cellular signaling network by interacting with a plethora of host proteins, and it is perplexing in its promiscuity. However, classifying these targets based on function reveals that Nef associates mostly with proteins involved in TCR signaling [7–9] and in trafficking of cell-surface receptors such as MHC proteins and CD4 [10–18]. Down modulation of membrane CD4, the HIV receptor, by Nef, prevents super-infection [18] and increases the budding efficiency of the HIV particle [19,20]. Moreover, downregulation of MHC from the plasma membrane by Nef protects the infected cells from killing by cytotoxic T lymphocytes[21]. These functions of Nef are theorized to contribute to the pathogenesis of HIV/AIDS.

β -catenin (β -cat)/Armadillo (*arm*), a component of plasma membrane juxtaposed adherens junctions is a key regulator of the evolutionarily conserved Wnt/*wingless* (*wg*) signaling pathway [22]. Activation of the canonical Wnt pathway leads to the stabilization of the cytoplasmic pool of β -catenin, which is otherwise phosphorylated by glycogen synthase kinase-3 β (GSK-3 β) and subsequently degraded by the ubiquitin-proteosome pathway [23]. β-catenin is known to act as a transcription factor by forming a complex with the LEF/TCF (Lymphoid Enhancer Factor/TCell Factor) family of HMG-box (high mobility group) transcription factors [24,25]. Upon Wnt stimulation, stabilized β-catenin translocates to the nucleus, where, together with LEF/TCF transcription factors, it activates downstream target genes [26]. 

β-catenin has numerous effects on T-cell development but its function in mature T-cells has only recently come to light. Studies in primary human T-cells have shown that β-catenin expression is upregulated rapidly after T-cell receptor (TCR) stimulation [27]. Disruption of the β-catenin-TCF interaction via ICAT expression impairs survival of thymocytes and activated mature T-cells [28]. In model systems and cells, β-catenin is known for its direct effect on cytoskeleton rearrangement, so it is not surprising that recent work demonstrates its involvement in T-cell extravasation in mature T-cells [29]. In this paper, we tested the hypothesis that Nef is a ligand of β-catenin. We show that Nef is structurally compatible with β-catenin, and that the interaction takes place *in vitro* and in cells. 

## Results

### The sequence pattern corresponding to the three-dimensional structural motif for β-catenin binding identifies HIV-Nef as a potential β-catenin ligand

**Figure 1:**
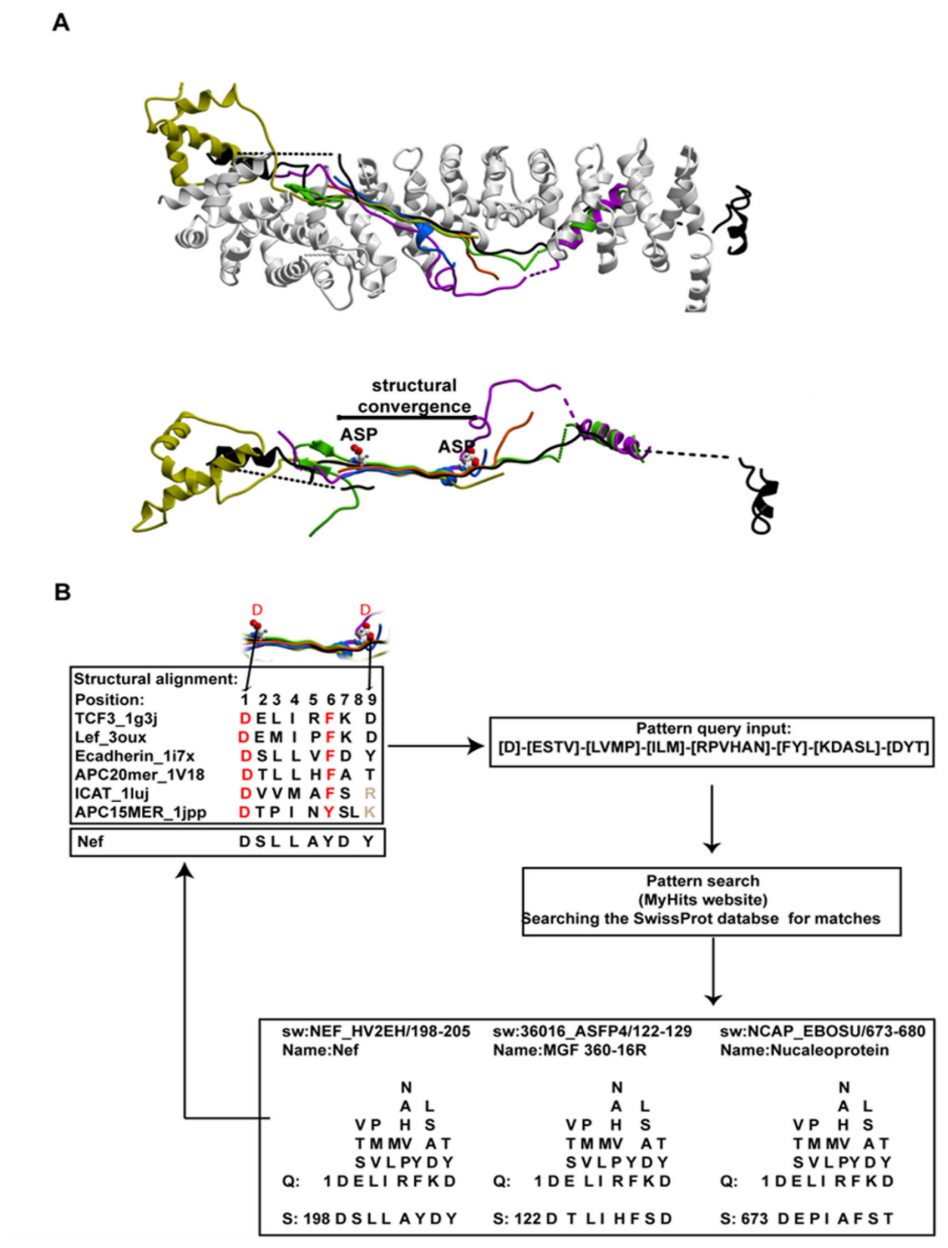
β-catenin ligands and the identification of Nef as a novel ligand. A. Upper panel: superimposition of known ligands of β-catenin as bound to β-catenin. β-catenin is shown in white, TCF3 is shown in green, ICAT is shown in yellow, LEF is shown in magenta, E-cadherin is shown in black, APC20 mer is shown in orange and APC15mer is shown in blue. Lower panel: same as the upper panel, however, β-catenin is not displayed. The region where the ligands converge to the same conformation is marked with a black line and titled “structural convergence” in the diagram. The two aspartates of TCF3 at the margins of the convergence are shown in x-stick representation. This region was used as the structural alignment of the ligands for the purpose of deriving the motif illustrated in panel B. B. Flow chart of the steps to identify Nef as a candidate β-catenin ligand. The chart begins with a structurally determined multiple alignment that was used to derive the β-catenin binding pattern motif. The most important residues for binding to β-catenin are colored in red. In grey, residues that were excluded from the motif as they don’t occupy the same position in 3D bound to the receptor (arginine from ICAT and lysine from APC 15 mer). Next, a Prosite-style sequence pattern was constructed to reflect the 3D structural pattern of compatibility of each residue at each specific location in the β-catenin ligand. For example, position 4 in the central region exhibits an isoleucine in some ligands and a leucine or methionine in others in contact with a hydrophobic patch on β-catenin. Thus, any of these three side chains may occupy this position and this portion of the pattern was defined as “[ILM]” to reflect this characteristic. The derived pattern then served as an input for the “MyHits” website that identified the above motif within the Nef, nucleocapsid and the MGF proteins.

Inspection of several β-catenin co-crystal structures (pdb code: 1g3j-complexed with TCF3 , 3oux-complexed with LEF1, 1i7w-complexed with E cadherin, 1v18-complexed with APC 20MER repeat, 1luj-complexed with ICAT and 1jpp-complexed with APC 15MER repeat) reveals that all ligands vary in structure [30–35]. However, a similar segment in all of these ligands adopts a homologous extended backbone structure at the center of their interface with β-catenin, with conformational variability of the periphery (Figure 1A). Structure-based alignment of the ligands (Figure 1B), focusing on the region where they all adopt a similar structure (Figure 1A, lower panel), was used to derive a sequence pattern (motif) for β-catenin binding. The motif, in Prosite notation (i.e. http://prosite.expasy.org/prosuser.html), is: [D]-[ESTV]-[LVMP]-[ILM]-[RPVHAN]-[FY]-[KDASL]-[DYT]. This motif captures the precise three-dimensional structural profile specific for the central region of β-catenin binding. Notably, the first position of the pattern is restricted to aspartate as this side chain is snugly buried in a highly specific β-catenin surface pocket and no substitutions are seen in any of the bound ligands. The same is true for the sixth position where the phenylalanine or tyrosine occupy a slot-like pocket restrictive for a benzene aromatic ring. The motif was then used as an input for a MyHits “pattern search” (http://myhits.isb-sib.ch/cgi-bin/pattern_search) [36] of the SwissProt database for novel viral ligands of β-catenin. 

The search specifically identified three potential β-catenin ligands: Nef, MGF 360-16R (a putative African Swine Fever Virus (ASFV) protein) and nucleocapsid protein (NCAP_EBOSU) (Figure 1B; Data File S1). Intriguingly, both Nef and the MGF 360 DNA region of ASFV are important for efficient viral growth in the host [37,38], however the MGF 360 DNA region is simply an open reading frame and the existence of a real, expressed protein is uncertain. We thus decided to focus on the hypothesis that Nef is a previously unrecognized β-catenin ligand. 

**Figure 2:**
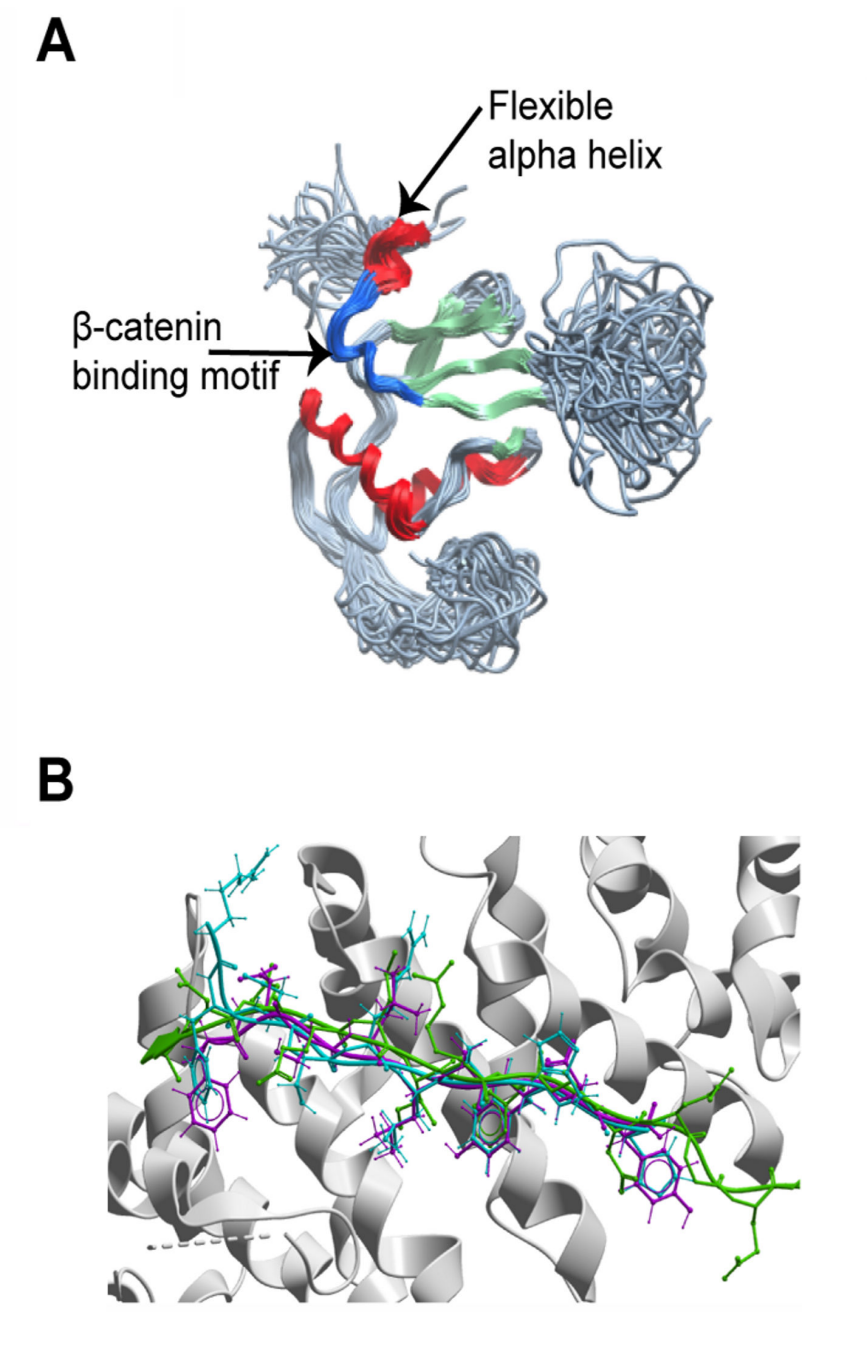
Structure and sequence based evaluation of Nef. A. Superimposition of all 40 NMR structures of Nef (pdb code 2nef). The proposed β-catenin binding motif is colored blue. B. Docking of HIV1 and HIV2 Nef peptides (shown in cyan and magenta, respectively) containing the motif to β-catenin binding (shown in white). Location of the β-catenin ligand, TCF3 (shown in green), from the high-resolution crystallographic structure of the complex (pdb code: 1g3j).

Initial structural analysis of the specific segment in question did not rule out the interaction. The Nef sequence corresponding to the β-catenin motif (DSLLAYDY) is very similar to the β-catenin binding sequence of E-cadherin (DSLLVFDY) (Figure 1B). In addition, the HIV-1 Nef NMR structures (pdb code: 2nef, [39]) reveal that the putative β-catenin binding fragment is accessible on the surface of the protein, albeit in a different conformation from that adopted by the equivalent segment in E-cadherin bound to β-catenin (Figure 2A). The NMR structures show that the β-catenin binding fragment is located in a particularly flexible region, adjacent to a flexible alpha helix (Figure 2A), suggesting that there is no significant barrier to rearrangement of this segment into the β-catenin binding conformation. Thus, the specific segment of Nef predicted to interact with β-catenin is not structurally incompatible with the ligand-binding site on β-catenin.

If Nef is indeed a true ligand of β-catenin, the key amino acids in the β-catenin motif should be conserved across diverse viral strains. Alignment of endogenous β-catenin ligands reveals that only D186 and F or Y at position 191 are conserved among all the ligands: alanine scanning of APC, TCF4 and E-cadherin showed that these key residues have the strongest effect on β-catenin binding [40]. Thus, the most sensitive motif for β-catenin binding is D-x-x-x-x-[FY] (This differs from the most specific motif we used to detect the interaction in the first place). This motif is conserved amongst Nef proteins from diverse viral strains, however it is poorly conserved in HIV subtypes A, C and D, while it is almost universally present in subtype B strains (Table 1). 

**Table 1:** Percentage of Nef sequences containing the β-catenin binding motif, D-x-x-x-x-[FY].

**Virus**	SIV	HIV-2	HIV-1	HIV-1	HIV-1	HIV-1
**Subtype**	n/a	n/a	A1	B	C	D
**Number of strains in LANL**	165	39	184	1880	652	105
**Percentage bearing D-x-x-x-x-[FY]**	60%	58.3%	19.8%	92.7%	2.6%	9.3%

Nef sequences from SIV, HIV2 and HIV1 subtypes A1-D were downloaded from the HIV LANL database, with only one sequence per patient selected. The motif D-x-x-x-x-[FY] is depicted in Prosite style (http://prosite.expasy.org/prosuser.html), where D stands for Asp residue in first position (position 186 in Nef), followed by any other four residues and the segment ends with either a Phe or a Tyr at position 191.

### Nef peptides docked to β-catenin exploit the same structural motifs as the known β-catenin ligands

The three-dimensional structural compatibility of Nef peptides with the β-catenin ligand-binding site was tested more stringently by computational molecular docking (ICM-DOCK; Molsoft, LLC, La Jolla CA). The peptides RFDSRLAFHH and FDSLLAYDY containing the β-catenin motif from NA7-Nef from HIV-1 and Nef- HV2EH from HIV-2, respectively, were docked to the molecular surface of the 3D structure of β-catenin. The 3D structure of β-catenin from the E-cadherin/β-catenin crystal structure (pdb code 1i7w) was chosen as the receptor, since Nef is similar in sequence to E-cadherin. Although the peptides were completely unconstrained with respect to location and conformation in the docking simulation, the Nef peptides preferentially docked into β-catenin’s ligand binding groove in a conformation very similar to E-cadherin with the lowest calculated energy out of theoretically billions of alternative conformations and alternative docking locations all over β-catenin’s surface (Figure 2B). As the search was not biased to this location on the structure, the docking results suggest that this fragment of Nef has a strong and exclusive conformational preference for the β-catenin ligand binding site, and that the preference is for the same orientation and atomic contacts observed in known β-catenin ligands. The probability of such an occurrence by random chance is very low [41] even for a sequence with similarity to β-catenin ligands. The contact areas of Nef residues in the β-catenin binding motif upon docking to β-catenin are summarized in Table S1.

### Nef interacts with β-catenin *in vitro*

**Figure 3:**
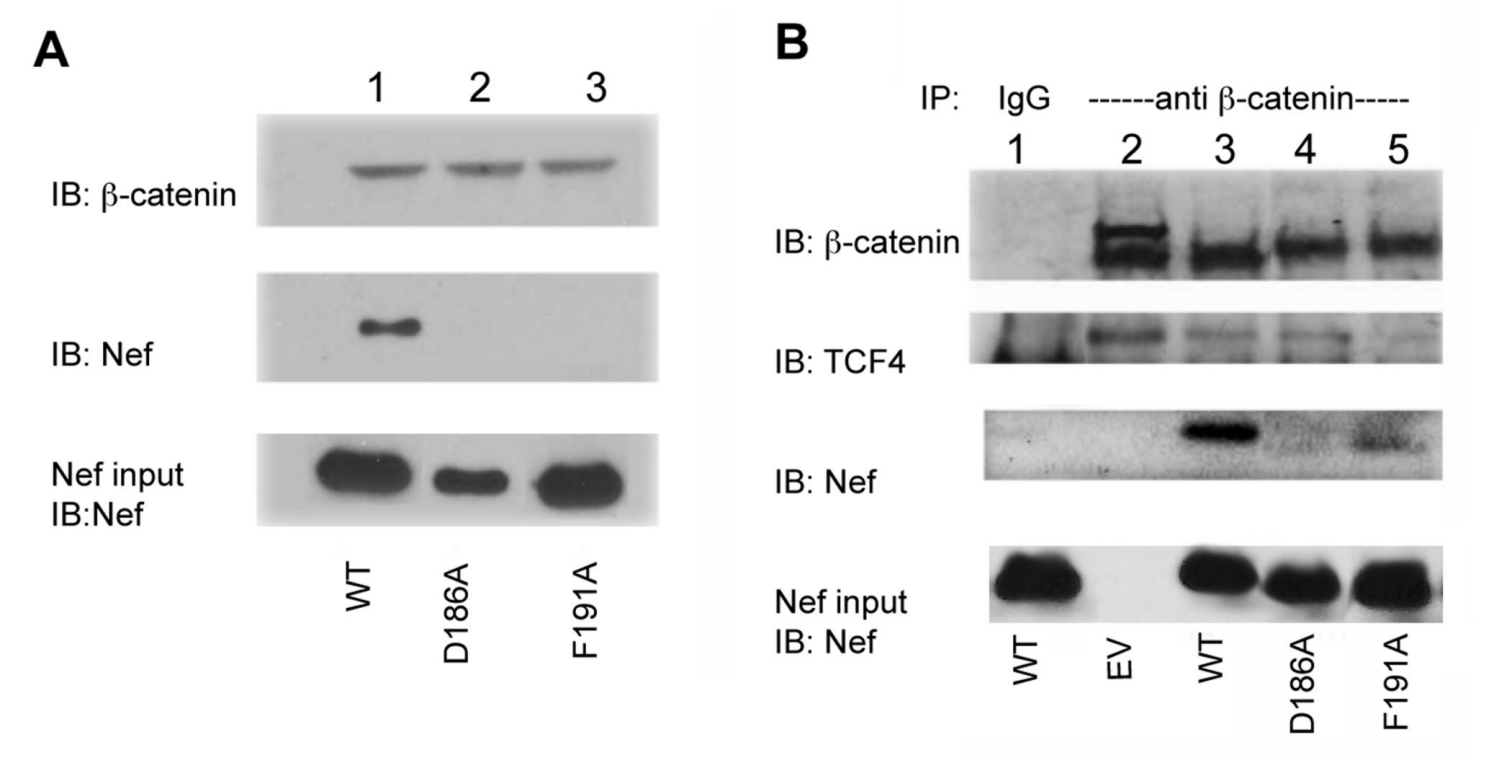
Nef interacts with β-catenin. A. Interaction of Nef and β-catenin *in* *vitro*. Purified recombinant WT-GST-Nef (or indicated D186A/F191A mutants), His- β-catenin and nickel beads mixed in physiological buffer, washed and eluted (See Methods). Top panel: immunoblot using mouse anti- β -catenin antibody (Ab) for detection. Middle panel: immunoblot using mouse anti-GST Ab for detection of Nef. Bottom panel: immunoblot using mouse anti-GST Ab for detection of Nef in total *E. coli* expression extract (input). This experiment was repeated three times and one representative immunoblot is shown here. B. Interaction of Nef and endogenous β-catenin in cells. HEK293 cells expressing wild-type (WT), the indicated mutants of Nef or empty vector (EV) by transfection are lysed and co-immunoprecipitated using a mouse anti- β-catenin Ab. Uppermost panel: immunoblot of immunoprecipitate (IP) using mouse anti- β-catenin Ab for detection. 2^nd^ to top panel: immunoblot of IP using anti-TCF4 Ab for detection. 3^rd^ to top panel: immunoblot of IP using mouse anti-Nef Ab for detection. Lowest panel: immunoblot of the total cell lysate prior to IP (input) using mouse anti-Nef Ab showing level of Nef (or mutant Nef) expression. This experiment was repeated three times and one representative immunoblot is shown here.

The interaction of wild-type Nef (WT-Nef) and wild-type β-catenin was confirmed *in-vitro* by a pull-down assay using purified recombinant proteins (Figure 3A). The observed interaction does not appear to be due to non-specific binding to beads or GST (Figure S1). While wild-type GST-Nef (WT-GST-Nef) elutes together with β-catenin (Figure 3A, lane 1), the D186A and F191A mutations abrogate the binding (Figure 3A, lane 2 and 3). This suggests that Nef directly interacts with β-catenin and that aspartic acid and phenylalanine, in positions 186 and 191 respectively, are hotspots of the interaction *in vitro*, as they are for other β-catenin ligands. 

### Interaction between β-catenin and Nef in HEK293 cells

We used co-immunoprecipitation (co-IP) experiments to determine whether Nef and β-catenin interact in a cellular context. HEK293 cells with endogenous β-catenin, expressing Nef via transfection, were lysed with mild detergent n-octyl glucoside lysis buffer followed by incubation with anti-β-catenin antibody. Co-IP of TCF4 and β-catenin serves as a positive control, since TCF4 is a known ligand of β-catenin (Figure 3B, lanes 2-5, second to top panel). Incubation with Protein A beads and washing demonstrated that WT-Nef specifically associated with β-catenin in cells (Figure 3B, lane 3). D186A-Nef did not associate with β-catenin, and F191A-Nef appeared at reduced levels (Figure 3B, lanes 4 and 5). When cells are not transfected with Nef, the TCF4 interaction is detected and Nef is not detected by western blot (Figure 3A, lane 2). IgG binds neither β-catenin, TCF nor Nef (Figure 3A, lane 1). The results demonstrate that endogenous β-catenin specifically co- precipitates with the WT-Nef construct. Fractionating Nef expressing HEK293 cells shows that WT-Nef is localized almost entirely in the cytoplasm (Figure S2). We therefore conclude that in HEK293 cells, the interaction we detected occurs in the cytoplasm.

### Nef inhibits the Wnt signaling pathway in HEK293 cells

**Figure 4:**
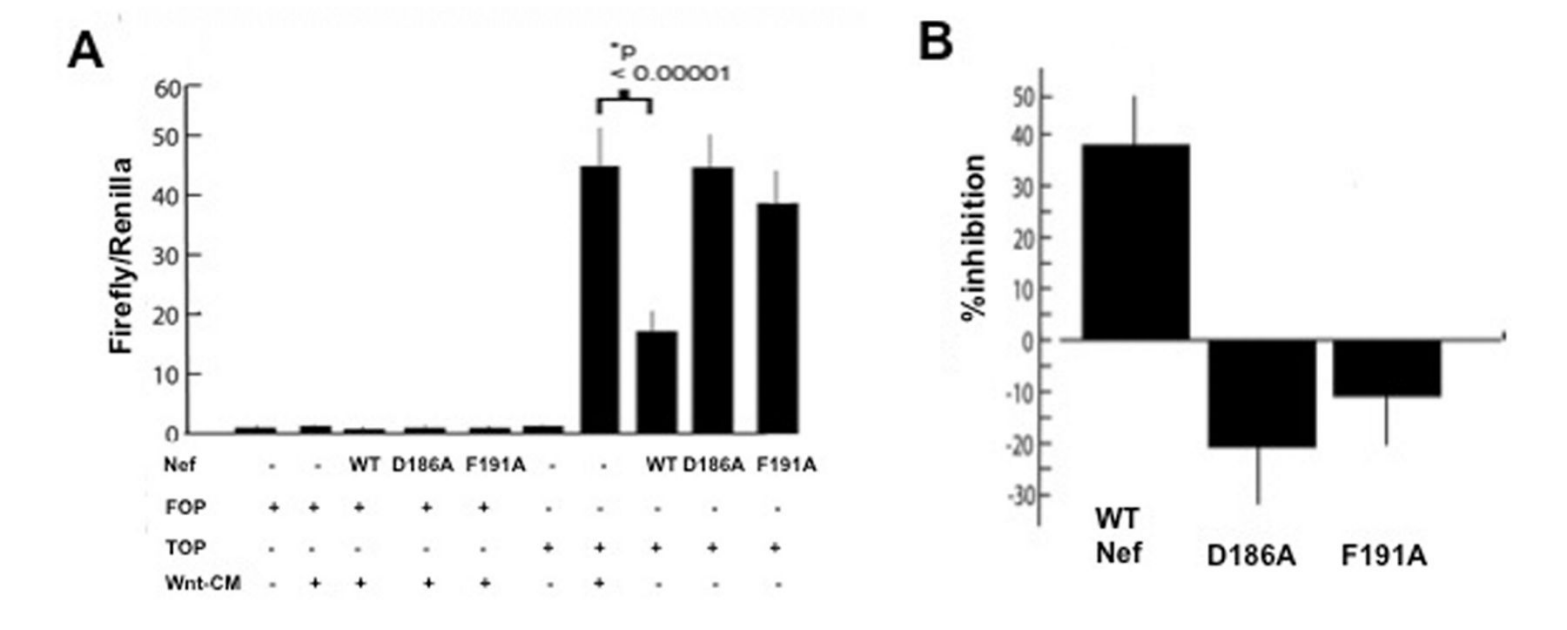
Nef inhibits Tcf –luciferase reporter activity. A. Promoter negative control: HEK293 cells were transfected with the TopFlash or the FopFlash reporters and with 50 ng Nef encoding plasmid (WT-Nef, the indicated mutants or empty vector) and incubated with Wnt-conditioned media (Wnt-CM) or control media that contain no Wnt. The TopFlash plasmid has TCF binding sites that are mutated in the FopFlash plasmid. The sites in FopFlash do not interact with TCF and therefore FopFlash serves as a negative control. Renilla luciferase was used for normalization purposes. Data shown here represent two experiments, each done in triplicate. WT-Nef significantly (P<0.00001, t-test) inhibits TCF reporter activity as compared to empty vector and also compared to the β-catenin motif mutants, D186A-Nef and F191A-Nef (P<0.00001, t-test) when co-transfected with TopFlash. B. The firefly/renilla values from panel A of this figure were used to calculate the TopFlash/FopFlash ratios of each condition. The resultant TopFlash/FopFlash ratios were then used to calculate the percent inhibition by WT-Nef and the indicated mutants on transcription in cells stimulated with Wnt.

To determine the activity of Nef in the Wnt signaling pathway, we measured its effect on the TopFlash plasmid, which is a β-catenin responsive luciferase reporter containing TCF-binding sites for TCF in human HEK293 cells. To verify that the observed effect is indeed β-catenin dependent, the effect of Nef on transcription from a FopFlash was assayed as well. The FopFlash plasmid has mutations in the TCF binding sites and therefore is not responsive to Wnt/ β-catenin signaling. Transfection of Nef significantly inhibited TopFlash reporter activity (P<0.00001, t-test), and the β-catenin motif mutants restored reporter activity to near control levels (Figure 4A). FopFlash reporter activity was not significantly altered with any of the Nef construct transfections, supporting the view that the observed effect of Nef on reporter activity was β-catenin dependent. Reporter activity was estimated to be inhibited by about 40% in the HEK293 cells (Figure 4B).

## Discussion

We report convergent structural, biochemical and cell biological observations supporting the conclusion that HIV’s Nef protein interacts with human β-catenin. A sequence near the C-terminus of Nef is similar to the central β-catenin binding motif in diverse β-catenin ligands. Isolated peptide structures exhibiting this sequence and its variations in Nef have a high degree of 3D structural compatibility with, and indeed a biophysical preference for, the molecular surface pocket on β-catenin, in which known β-catenin ligands bind (shown by unconstrained computational molecular docking). The distribution of the β-catenin binding motif in Nef across diverse HIV-1, SIV and HIV-2 strains suggests conservation of the interaction site. The interaction is specifically detected *in vitro* and in HEK293 cells. Finally, the interaction is functionally significant in HEK293 cells: the endogenous Wnt signaling pathway in this human cell line is affected by Nef and this influence disappears when Nef positions 186 and 191, which mirror key residues in known β-catenin ligands, are mutated. 

The 3D structural and informatics results strongly suggest that the interaction is relevant for HIV/AIDS, as the motif appears across HIV strains, suggesting functional conservation in the virus. However, the HEK293 cell line in which we demonstrated the cellular activity is not directly relevant to HIV/AIDS. Nevertheless, endogenous human β-catenin was bound in these cells. This fact, along with the structural results, increases the probability that Nef may play a role in AIDS pathogenesis.

From a cell biology point of view, the probability that Nef interacts with β-catenin leads to intriguing speculation that β-catenin-mediated functions in T-cells and macrophages are involved in HIV pathophysiology. Nef is already known to be associated with T-cell chemotaxis, which is the driving force of T-cell extravasation to infected tissues [42–45]. Interestingly, β-catenin is involved directly in the physical process of T-cell extravasation: stabilized β-catenin protein in T-cells directly targets matrix metalloproteinase (MMP) promoters through tandem TCF sites and MMP expression augments T-cell transmigration [29]. In addition, β-catenin stabilization in T-cells enhanced survival of CD4+/CD25+ T-regulatory cells (Treg) while CD4+/CD25- T-non-regulatory cells (Tnon-reg) became anergic and proliferated poorly in response to CD3 antibody stimulus [46]. A mirroring pattern of immune activation appears to occur during HIV/SIV infection [47,48]. Finally, recent studies show that β-catenin signaling programs dendritic cells in the gut into a tolerogenic state, limiting the inflammatory response there [49]. The gut is the principal site where HIV-1 replicates [50–55].

If it is physiologically relevant, the β-catenin interaction site is predicted by Table 1 to be highly prevalent in HIV subtype B, SIV and HIV-2, while it is nearly absent in the other HIV subtypes. Although differences in anti-retroviral treatment confound analysis, there appear to be phenotypic differences between B and non-B HIV subtypes [56,57]. There are clear phenotypic differences between HIV-1 and HIV-2, as well as between SIV and HIV. Our findings raise the possibility that Nef’s interaction with β-catenin may contribute to phenotypic differences observed between B and non-B subtypes in HIV-1, and to the phenotypic differences between HIV-1, HIV-2 and SIV. If this were true, the exact contributions and specific phenotypes in question would require further investigation. 

How might Nef influence β-catenin signaling *in vivo*? At first approximation, our results suggest that Nef can compete for the same site occupied by TCF/LEF on β-catenin, thereby inhibiting TCF-based transcription (Figure 4). However, Nef is primarily (although not exclusively) localized to the cytoplasm [58,59], while the TCF- β-catenin complex is active in the nucleus of cells [24–26]. This raises the possibility, even though we have observed functional inhibition of TCF-based transcription, that Nef could affect β-catenin stabilization in the cytoplasm, compete with E-cadherin at cell-cell junctions [60] or even compete with ICAT [61] in the nucleus resulting in *increased* TCF-based transcription in T-cells or macrophages (which may be fundamentally physiologically different from the HEK293 cells used in this study). Fractionation studies performed in our lab in HEK293 cells (Figure S2) show that in this particular cell line, Nef localization is limited to the cytoplasm with only traces in the nucleus. Nevertheless, the predominant *in vivo* functional effect, if any, of Nef on T-cells and macrophages infected with HIV could diverge significantly from our observations in HEK293 cells.

Minor informatics improvements enabled our detection of the Nef-β-catenin interaction. Searching for new ligands to β-catenin with a motif, rather than using standard sequence-sequence alignment methods enabled us to identify new ligands. In 1993, Shugars et al. identified four Nef-defining sequences, referred to as “blocks”. The blocks were aligned with host proteins in order to shed light on the function of Nef. The C terminal block, overlapping with the β-catenin binding motif (Figure 1B), did not align with host proteins and its function remained unclear [62]. The use of a motif in contrast to *sequence-sequence* alignment is therefore more productive and this aligns with work done by others [63]. In addition, 3D structural information is encapsulated within our motif, which further increases the sensitivity of the motif.

**Figure 5:**
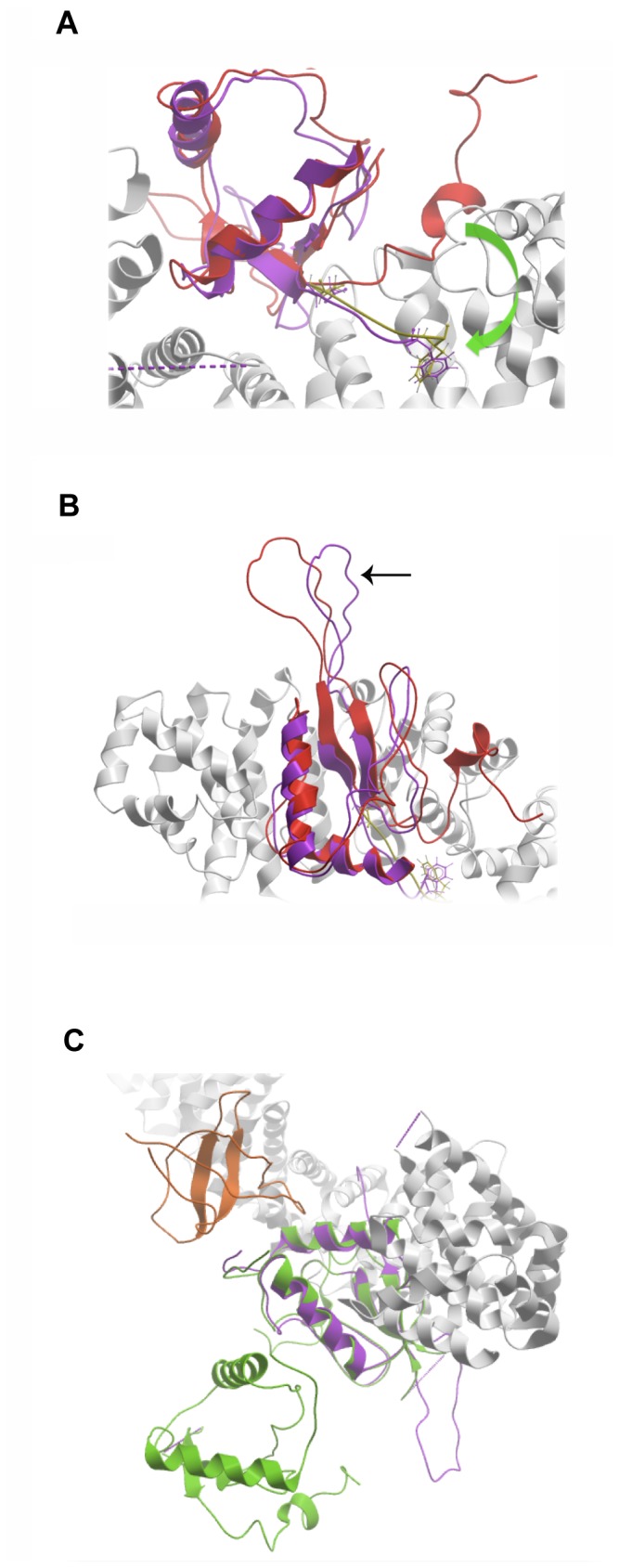
Model of Nef *in situ* on β-catenin. A. The C-terminal tail of Nef after position 185 is rearranged from its unbound position into the conformation seen in β-catenin ligands. The curved arrow shows the hypothesized trajectory of the rearrangement. The backbone of un-rearranged Nef is shown in red ribbon, re-arranged Nef in magenta, β-catenin in grey, bound E-cadherin in yellow. The key hotspots (Asp674/ Phe679 of E-cadherin and Asp186/Phe191 of Nef) are displayed in stick depiction. B. A different view of the complex described in 5A. The black arrow points to the loop in Nef that is important for binding to adaptor proteins, MHC proteins, and is also important in sorting these proteins into clathrin-coated pits ([10,15,85,86]). C. The β-catenin bound form of Nef (magenta) is superimposed with a dimeric Nef conformation (green) bound to the Fyn SH3 domain (orange; pdb 1avz, [87]).

Structurally, the match between Nef and β-catenin is very strong, with the local sequence of Nef at the location of the β-catenin motif easily adopting, and actually preferring, a structural conformation that fills the key Asp and Phe/Tyr restricted pockets on β-catenin (Figure 1). In addition, although the major hotspot of the interaction was determined to be this small segment of Nef at 186-191, Nef binds a variety of armadillo repeat proteins as a whole domain [10,11,64–70]. Accordingly, there may be other distributed contact points on the Nef and concave β-catenin surfaces that contribute to the interaction. Indeed, a nuclear receptor [71] is known to bind to β-catenin as a whole domain within the concave surface of its armadillo repeats. In order to visualize whether such an interaction could be consistent with our findings, we built a theoretical model of the whole Nef domain bound to β-catenin via the segment at 186-191. Our model shows that the C-terminal tail of Nef after position 185 must detach from the core Nef domain and unfold in order to assume the extended conformation predicted by our studies and make the key β-catenin interactions (Figure 5A). The remaining core domain almost perfectly fills the volume of the concave surface of β-catenin. A highly flexible loop in Nef that is important for its association with adaptor proteins (marked with an arrow in Figure 5B) does not clash with β-catenin in our model. Figure 5C reveals that, if Nef were to bind to β-catenin in this manner, it would not interfere with Nef dimerization or with other Nef binding sites such as that for the SH3 domain from Fyn.

The Wnt pathway is an intrinsic molecular mechanism to limit HIV replication in PBMC’s (peripheral blood mononuclear cells) and in astrocytes [72,73]. It was shown that HIV replication is repressed when TCF-4 transcription factor binds the HIV long terminal repeat (LTR)[73]. At least four such TCF-4 binding sites have been identified in the LTR. The -143 site has garnered attention since it (i) has 100 % homology to the TCF-4 core (5′-(A/T)(A/T)CAAAG-3′), (ii) it is present in approximately one-third of 500 HIV LTR sequences from the the Los Alamos gene bank, (iii) it has the highest affinity for TCF-4 (iv) SMAR1, a nuclear matrix binding protein, was shown to complex with β-catenin/TCF-4 at the -143 site to facilitate transcriptional repression from HIV promoters[74–76]}. In addition, it was shown that expression of TCF-4 in human astrocytic cells decreased the basal and Tat-mediated transcription of the HIV-1 LTR [77]. TCF-4/β-catenin repression of basal LTR activity likely prevents Tat from reaching a threshold level which would allow it to tether on the TAR region of the LTR in association with a positive elongation complex (pTEFb) to accelerate the rate and efficiency of HIV transcription[75].

TCF-4 and β-catenin regulate the expression of other transcription factors relevant to HIV transcription. β-catenin and TCF-4 inhibit C/EBP β/δ tethering on the HIV LTR, suggesting that β-catenin and TCF-4 cooperate in this repression. TCF-4, independent of β-catenin, also negatively regulates NFκB tethering on the LTR [78]. In astrocytes, NFκB suppression is mediated by TCF-4 without the involvement of β-catenin while in other cells, β-catenin suppresses NFκB activity [79]. TCF-4 suppression of NFκB may be mediated by direct interaction between TCF-4 and NFκB or an indirect effect on upstream regulators of NFκB. Both C/EBP and NFκB are inducers of HIV promoter activity and thus β-catenin/TCF-4 inhibition of these inducers could also contribute to the overall mechanism by which the Wnt/β-catenin pathway represses HIV transcription and replication.

These data correlate with our reporter assay results in which Nef inhibits transcription from a luciferase reporter with multiple TCF binding sites. Our results suggest that Nef prevents TCF-4 from repressing HIV replication via its action on β-catenin. Extrapolating our findings from HEK293 cells to immune cells *in vivo*, it is possible, though speculative, that the Wnt pathway may mediate the lowered viral load and lack of progression associated with Nef deletion [1,4,5,80].

We have detected a previously unrecognized interaction between the HIV-1 protein Nef and human β-catenin, part of the Wnt signaling pathway. This finding potentially implicates β-catenin and the Wnt signaling pathway in T-cell transmigration defects and immune activation phenomena observed during the development of AIDS from HIV infection. 

## Methods

### β-catenin binding pattern design

β-catenin co-crystal structures (pdb code: 1g3j, 3oux, 1i7w, 1v18, 1luj and 1jpp) [30–35] were superimposed using ICM [81]. Ligand sequences were then aligned based on the position of each residue in 3D space according to the above crystal structures. The pattern: [D]-[ESTV]-[LV]-[ILM]-[RSVHA]-[FY]-[KDAS]-[DYTS], derived from the structure based alignment, serves as a regular expression that restricts the acceptable amino acids for a given position, listing them between square parentheses '[ ]'. For example: [ESTV] stands for glutamic acid, serine, threonine or valine in the indicated position. The sequence pattern was then used as an input for the “pattern search” service that is integrated in the MyHits website the (http://myhits.isb-sib.ch/cgi-bin/pattern_search) to identify new β-catenin ligands within the SwissProt database, while restraining the taxonomic range to Viruses [taxid: 10239] [36].

### Nef sequence analysis

Nef sequences from SIV, HIV2 and HIV1 subtypes were downloaded from the HIV Sequence Alignment tool incorporated in the HIV LANL database, with only one sequence per patient selected (http://www.hiv.lanl.gov/content/sequence/NEWALIGN/align.html). The percentage of Nef sequences carrying the D-x-x-x-x-[FY] motif were calculated with a custom Perl script (available upon request from the authors).

### Docking

Crystal structures of the ARM repeat of β-catenin in complex with E-cadherin (pdb code: 11i7w) were used for the docking simulation. Hydrogen atoms were added to the crystal structure and the structure was then converted into an internal coordinate representation according to the ICM method [82]. Full-atom models of the peptides “RFDSRLAFHH” and “FDSLLAYDY” were built in the same internal coordinates. The peptide/β-catenin pairs in the set were docked using the Biased-Probability Monte Carlo conformational search algorithm to search all of the conformations of the full-atom model of the peptide within the space of grid potential maps calculated from the β-catenin receptor structure coordinates as implemented in the ICM software [83].  Van der Waals, electrostatics, entropy and hydrogen bonding energy terms were evaluated during the search.  

### Nef protein purification

*Escherichia coli* BL21 (DE3) harboring pGEX-4T-2-NEF (NA7, SwissProt accession: Q306M3) (a gift from Prof. Steven Burakoff, Mount Sinai School of Medicine) were grown in LB medium containing 100 μg/ml ampicillin at 18 °C. At the exponential phase of growth (OD600=0.6), isopropyl- β -Dthiogalactopyranoside (IPTG) was added to the culture medium at a final concentration of 0.8 mM. After an additional 12 hours of culture, cells were harvested by centrifugation (4200 rpm for 15 minutes). The bacterial pellet was suspended in lysis buffer (300mM NaCl, 50mM TrisHcl pH8, 10% glycerol, protease inhibitors, 1mM PMSF, 2M urea, 1% triton, 5mM DTT, 1mM EDTA), sonicated on ice and centrifuged at 4 °C, 13,200 rpm for an additional hour. The supernatant was incubated for 1.5 hours with glutathione beads. The beads were washed three times with wash buffer (PBS supplemented with 200mM NaCl, 50mM TrisHcl pH8, 10% glycerol, 1mM PMSF, 2M urea, 1% triton,5mM DTT, 1mM EDTA) and an additional fourth wash (250 mM NaCl, 25mM TrisHcl pH8, 10% glycerol, 1mM PMSF, 2M urea,1% triton, 0.5M urea, 20mM β-mercaptoethanol). The beads were eluted with 50mM glutathione buffer (250 mM NaCl, 25mM TrisHcl pH8, 10% glycerol, 1mM PMSF, 2M urea, 1% triton, 0.5M urea, 20mM β-mercaptoethanol, 10mM imidazole).

### His-tagged β-catenin protein purification

*Escherichia coli* BL21 (DE3) harboring pExHTB-β-catenin (kindly provided by Prof. Bill Weiss, Stanford University) were grown and induced as described above. The cell paste was then suspended in lysis buffer (1% triton, 300mM NaCl, 50mM TrisHcl pH8, 10% glycerol, EDTA free protease inhibitors, 1mM PMSF, 15Mm imidazole, 20mM β-mercaptoethanol), followed by sonication on ice and centrifuged at 4 °C, 13,200 rpm for an additional hour. The supernatant was incubated for 1.5 hours with Ni beads. The beads were washed three times with wash buffer (250mM NaCl, 50mM TrisHcl pH8, 10% glycerol, 1mM PMSF, 1% triton, 20mM β-mercaptoethanol, 10mM imidazole) and an additional fourth wash (300mM NaCl, 50mM TrisHcl pH8, 10% glycerol, 1mM PMSF, 1% triton, 20mM β-mercaptoethanol, 10mM imidazole). For the purpose of the pull down assay, β-catenin was not eluted off the Ni beads.

### In-vitro binding assay

Purified GST-Nef was added to β-catenin that was immobilized on Ni beads as described above. The proteins were incubated together for 2 hours and washed three times with wash buffer (250mM NaCl, 25mM TrisHcl pH8, 1% glycerol, 1mM PMSF, 1% triton, 15mM β-mercaptoethanol, 10mM imidazole) and a fourth wash (300mM NaCl, 25mM TrisHcl pH8, 1% glycerol, 1mM PMSF, 1% triton, 15mM β-mercaptoethanol, 10mM imidazole). Beads were eluted with 250mM NaCl, 25mM TrisHcl pH8, 1% glycerol, 1mM PMSF, 1% triton, 15mM β-mercaptoethanol, 600mM imidazole) for 1h. Protein complexes bound to the beads were eluted and denatured by the addition of SDS. The samples were then loaded on a 10% SDS denaturing gel and transferred overnight into a nitrocellulose membrane. Proteins were detected by a western blot. β-catenin was detected by using a monoclonal mouse antibody (Sigma, clone 15B8). GST-Nef was detected by using a rabbit polyclonal rabbit anti GST antibody (Santa-Cruz, sc-8334). Goat peroxidase-conjugated anti-rabbit or anti-mouse antibodies (1:1000, R&D Systems) were used and binding was detected by enhanced chemiluminescence (ECL, Pierce). The experiment was repeated three times and a representative blot is shown in Figure 3A.

### Cell Culture

HEK293 cells were cultured in Dulbecco’s Minimal Essential Medium (Invitrogen), supplemented with 10% fetal bovine serum and sodium pyruvate. Cells were incubated in a 37°C incubator in an atmosphere of 5% CO_2_.

### Co-immunoprecipitation assay

Expression vectors encoding WT-Nef, D186A-Nef and F191A-Nef were transfected into 293 cells in 10cm plate with Effectene (Qiagen). The expression vector was obtained through the NIH AIDS Research and Reference Reagent program from Drs. Yingying Li, Feng Gao, and Beatrice H. Hahn (Cat# 6173). Cells were incubated for 16-18 hours post-transfection, washed 4 times with cold phosphate buffered saline (PBS), lysed with 1ml lysis buffer (180mM NaCl, 50mM TrisHcl pH7.5, 1mM PMSF, 0.5% n-Octylglucoside (roche), 1mM Na3VO4, 5mM EDTA, 1mM NaF, protease inhibitor) and put on ice for 2 hours. Cell suspensions were centrifuged for 10 minutes at 13,200 rpm, and the supernatants were incubated with monoclonal anti-β-catenin antibody (sigma, monoclonal clone 15B8) or with normal mouse IgG (Santa-Cruz). After 16-20 hours, 25 μl of protein A/G beads (Santa-Cruz) were added to the antibody-cell suspension solution for an additional 2 hours. Beads were washed with wash buffer (220mM NaCl, 50mM TrisHcl pH7.5, 1mM PMSF, 0.5% n-octylglucoside) four times by inverting the tubes three times followed by 5 minute incubation on ice and another three washes. 100μl of 3X sample buffer was added to beads and incubated on ice for 1.5 hours. Beads were boiled for 5 minutes and supernatants were resolved on a 12% acrylamide gel by electrophoresis at 90V. Gels were presoaked in transfer buffer (Tris, Glycine, 0.015% SDS, 20% Methanol) for 30 minutes at 4 degrees C and then transferred to nitrocellulose membranes (30V, 16 hours) using a transfer cell (Bio-Rad). 

### Immunoblotting

Nitrocellulose membranes were blocked with 5% fat-free dried milk in TBST (50 mM TrisHcl, pH 7.4, 150 mM NaCl, 0.1% Tween 20) for one hour at room temperature (RT). Membranes were incubated with primary antibody (monoclonal mouse β-catenin antibody obtained through sigma #C7207, TCF4 antibody obtained through Millipore #05-511) diluted in 2% milk in TBST (1:1000 dilution) for one hour at RT, followed by three washes with TBST for 10 minutes each. Secondary HRP antibodies (R&D) were then added (1:1000 dilution) one hour at RT with gentle agitation on a rocking plate. The signal was detected using ECL reagents (Pierce).

ONE-HOUR IP-Western Kit (Genscript) that specifically blocks light and heavy chain contamination in IP experiments was used to detect Nef. Nef antibody #1539 (obtained through the NIH AIDS Research and Reference program, Division of AIDS, NIAID, NIH from Dr. Kai Krohn and Dr. Vladimir Ovod [84]) was used in combination with this kit. The co-immunoprecipitation experiment was repeated three times and a representative blot is shown in Figure 3B.

### Luciferase assay

293 cells were transfected with 15ng TopFlash or FopFlash, 5ng CMV-Renilla and with either empty vector or 50ng of codon-optimized WT-Nef, or Nef mutants. The transfection mix was first added to each well in the 96-well plate and only then 293 cells were added to the plate. The FopFlash plasmid contains TCF binding sites which are mutated in the FopFlash plasmid. Transcription from this reporter plasmid is activated when β-catenin enters the nucleus and binds TCF. The TCF/β-catenin complex then binds to the TCF binding sites on the reporter to initiate transcription of Firefly luciferase. Cells were treated with Wnt-conditioned media to activate the Wnt pathway. 5 hours post transfection, cells were replenished with fresh Wnt-conditioned media for an additional 10 hours. The assay was performed by using the Promega Dual-Luciferase® Reporter Assay System. It is important to note that that WT-Nef and the two mutants affect Renilla’s trancription levels to the same degree (Figure 4B; Data File S2).

## Supporting Information

Data File S1 **The file documents the output of the “pattern search” tool implemented in the MyHits website (http://myhits.isb-sib.ch/cgi-bin/pattern_search).** The β-catenin binding motif is [D]-[ESTV]-[LVMP]-[ILM]-[RPVHAN]-[FY]-[KDASL]-[DYT] was used as an input to search for viral proteins in the SwissProt database containing the motif.
(PDF)

Data File S2 **Row data for luciferase assay in which 293 cells were transfected with 15ng TopFlash or FopFlash, 5ng CMV-Renilla and with either empty vector or 50ng of codon-optimized WT-Nef, or Nef mutants.**
(XLS)

Figure S1 **Coomasie Blue staining of three different pull down control experiments.** A. Pull down experiments of His-β-catenin and WT-Nef. “w” stands for washing step and “EL” stands for elution B. pull down experiment of His-β-catenin and GST C. Incubation of WT-Nef-GST and Ni beads.
(TIF)

Figure S2 **Cytoplasmic and nuclear fractionation of HEK293 cells transfected with different amounts of WT-Nef.** HEK293 cells were plated in a 10cm dish and transfected 24h later with an empty vector or WT-Nef encoding plasmid up to 2ng. The cells were lysed 15 hours post transfection. The nucleus (lanes 1-5) and cytoplasm (lanes 6-10) were extracted using the NE-PER kit by Thermo-Scientific. The cytoplasmic and nuclear fractions were blotted with anti-tubulin, a cytoplasmic marker and anti- Histone H1, a nuclear marker, in order to assess the purity of the cytoplasmic and nuclear lysates. The lysates were also blotted with anti-actin antibody as a loading control. Uppermost panel: immunoblot of endogenous β-catenin using mouse anti- β-catenin Ab for detection. 2^nd^ to top panel: immunoblot of different amounts of transfected WT-Nef (0-2ng) using mouse anti-Nef Ab for detection. 3^rd^ to top panel: immunoblot of alpha-tublin using anti-alpha tubulin Ab for detection. Lowest panel: immunoblot of actin using anti-pan-actin for detection.
(TIF)

Table S1 **Contact areas of Nef residues in the β-catenin binding motif upon docking to β-catenin.**
(DOC)
