# Supplementary material for: HIV’s Nef Interacts with β-Catenin of the Wnt Signaling Pathway in HEK293 Cells
Source: PLoS One. 2013 Oct 10;8(10):e77865. doi: 10.1371/journal.pone.0077865 (PMC3795062; doi:10.1371/journal.pone.0077865)
Supplement: Data File S1 — The file documents the output of the “pattern search” tool implemented in the MyHits website (http://myhits.isb-sib.ch/cgi-bin/pattern_search). The β-catenin binding motif is [D]-[ESTV]-[LVMP]-[ILM]-[RPVHAN]-[FY]-[KDASL]-[DYT] was used as an input to search for viral proteins in the SwissProt database containing the motif. (PDF) [file pone.0077865.s001.pdf]

Query Hub Result Help Database

text search

search help

user: GUEST

log in

Pattern Search Results

Pattern [D]-[ESTV]-[LVMP]-[ILM]-[RPVHAN]-[FY]-[KDASL]-[DYT].

Database of proteins Swiss-Prot [sw].

Taxonomic range Viruses [taxid:10239].

searching ...
postprocessing ...

Summary

Original output here

**Statistics**		this run	previous	gain	loss
	number of matches equal or below threshold	5	NA	NA	NA
	total	5	NA	NA	NA

Detail of matches

uncheck all | preselection | check all | Send checked matches to Please select

New

sw:NEF_HV2EH/198-205 .

motif=PATTERN RecName: Full=Protein Nef;AltName: Full=Negative factor; Short=F-protein; AltName: Full=3'ORF [entry]

```
                N
                A L
             VP H S
             TMMV AT
             SVLPYDY
Q:      1 DELIRFKD
             |:::::::
S:    198 DSLLAYDY
```

^ buttons ^

New

sw:NCAP_EBOSB/673-680 .

motif=PATTERN RecName: Full=Nucleoprotein;AltName: Full=Nucleocapsid protein; Short=Protein N [entry]

```
                N
                A L
             VP H S
             TMMV AT
             SVLPYDY
Q:      1 DELIRFKD
             |:::::::
S:    673 DEPIAFST
```

^ buttons ^

New

sw:NEF_HV2D2/200-207 .
motif=PATTERN RecName: Full=Protein Nef;AltName: Full=Negative factor; Short=F-protein; AltName: Full=3'ORF [entry]

```
              N
              A L
          VP  H S
          TMMV AT
          SVLPYDY
Q:     1 DELIRFKD
          |:::::::
S:   200 DSLLAYDY
```

^ buttons ^

New

sw:NCAP_EBOSU/673-680 .
motif=PATTERN RecName: Full=Nucleoprotein;AltName: Full=Nucleocapsid protein; Short=Protein N [entry]

```
              N
              A L
          VP  H S
          TMMV AT
          SVLPYDY
Q:     1 DELIRFKD
          |:::::::
S:   673 DEPIAFST
```

^ buttons ^

New

sw:36016_ASFP4/122-129 .
motif=PATTERN RecName: Full=Protein MGF 360-16R [entry]

```
              N
              A L
          VP  H S
          TMMV AT
          SVLPYDY
Q:     1 DELIRFKD
          |:::::::
S:   122 DTLIHFSD
```

^ buttons ^

MyHits © 2003-2011 **Question or comment about this page.**
