# Supplementary figures and images for: HIV’s Nef Interacts with β-Catenin of the Wnt Signaling Pathway in HEK293 Cells
Source: PLoS One. 2013 Oct 10;8(10):e77865. doi: 10.1371/journal.pone.0077865 (PMC3795062; doi:10.1371/journal.pone.0077865)

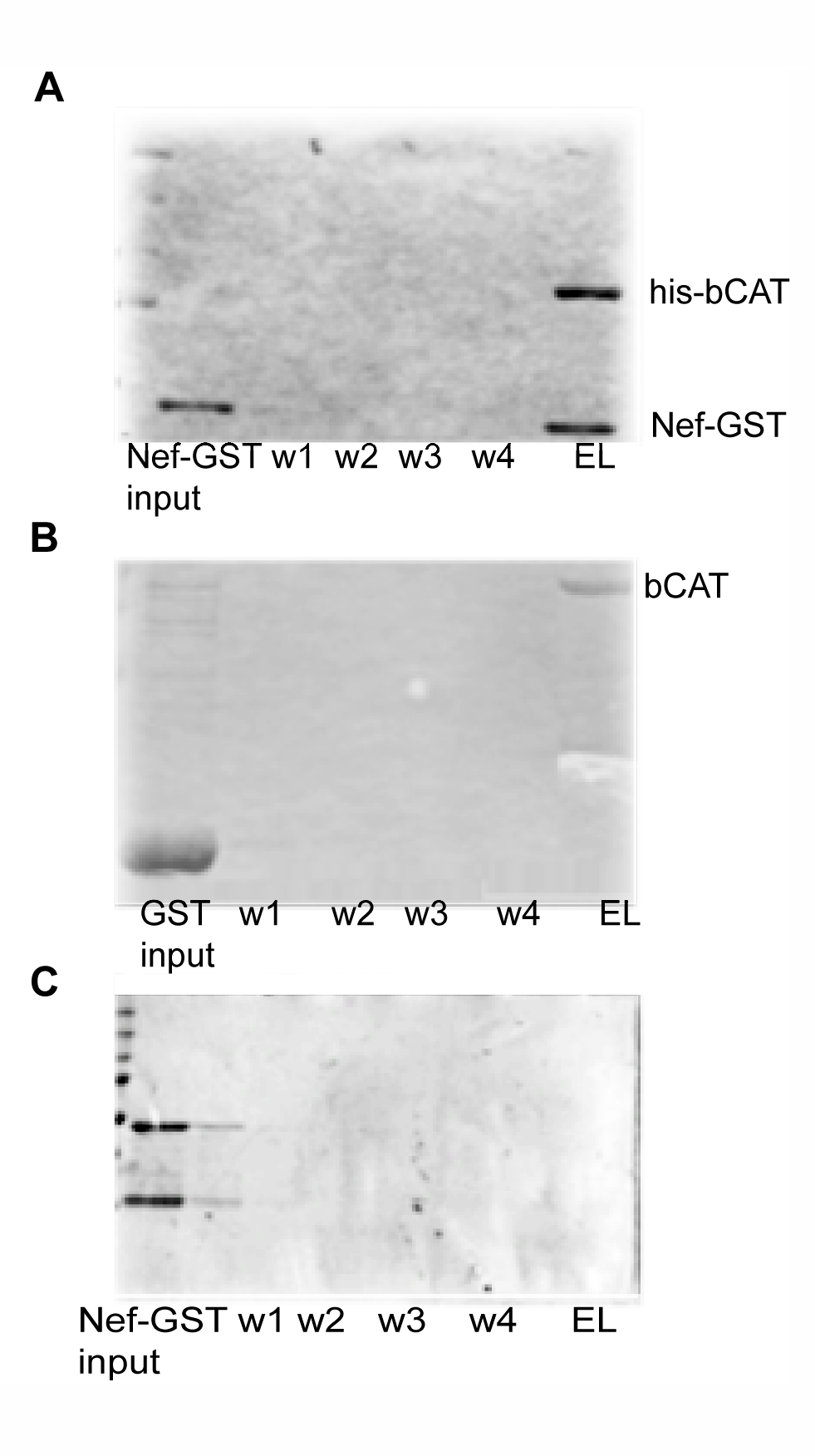

Supplement: Figure S1 — Coomasie Blue staining of three different pull down control experiments. A. Pull down experiments of His-β-catenin and WT-Nef. “w” stands for washing step and “EL” stands for elution B. pull down experiment of His-β-catenin and GST C. Incubation of WT-Nef-GST and Ni beads. (TIF) [file pone.0077865.s003.tif]

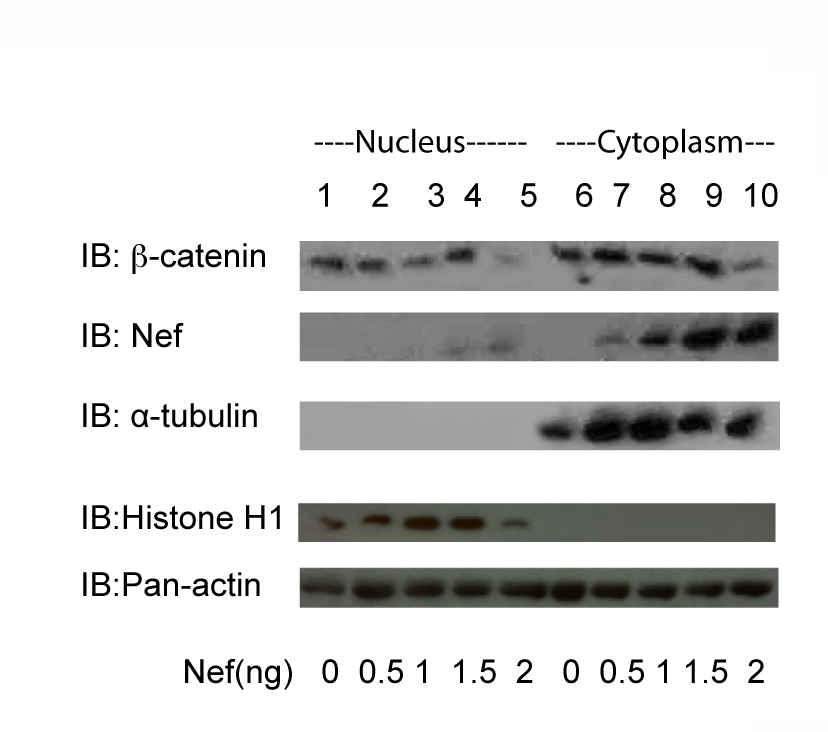

Supplement: Figure S2 — Cytoplasmic and nuclear fractionation of HEK293 cells transfected with different amounts of WT-Nef. HEK293 cells were plated in a 10cm dish and transfected 24h later with an empty vector or WT-Nef encoding plasmid up to 2ng. The cells were lysed 15 hours post transfection. The nucleus (lanes 1-5) and cytoplasm (lanes 6-10) were extracted using the NE-PER kit by Thermo-Scientific. The cytoplasmic and nuclear fractions were blotted with anti-tubulin, a cytoplasmic marker and anti- Histone H1, a nuclear marker, in order to assess the purity of the cytoplasmic and nuclear lysates. The lysates were also blotted with anti-actin antibody as a loading control. Uppermost panel: immunoblot of endogenous β-catenin using mouse anti- β-catenin Ab for detection. 2nd to top panel: immunoblot of different amounts of transfected WT-Nef (0-2ng) using mouse anti-Nef Ab for detection. 3rd to top panel: immunoblot of alpha-tublin using anti-alpha tubulin Ab for detection. Lowest panel: immunoblot of actin using anti-pan-actin for detection. (TIF) [file pone.0077865.s004.tif]
