# Supplementary material for: HIV’s Nef Interacts with β-Catenin of the Wnt Signaling Pathway in HEK293 Cells
Source: PLoS One. 2013 Oct 10;8(10):e77865. doi: 10.1371/journal.pone.0077865 (PMC3795062; doi:10.1371/journal.pone.0077865)
Supplement: Table S1 — Contact areas of Nef residues in the β-catenin binding motif upon docking to β-catenin. (DOC) [file pone.0077865.s005.doc]

| Residue  In HIV2 Nef | Contact Area (Å^2^) | Exposed  Area  (Å^2^) | Contact Area Percentage | Residue  In HIV1 Nef | Contact Area  (Å^2^) | Exposed  Area  (Å^2^) | Contact Area Percentage |
| --- | --- | --- | --- | --- | --- | --- | --- |
| HIV2 Nef peptide:  F^185^D^186^SLLAY^191^DY | | | | HIV1 Nef peptide: R^184^FD^186^SRLAF^191^HH | | | |
|  |  |  |  | R 184 | 62.8 | 226.7 | 28 |
| F 185 | 85.8 | 181.7 | 47 | F 185 | 50.6 | 185.0 | 27 |
| D 186 | 81.3 | 123.3 | 66 | D 186 | 83.3 | 108.8 | 77 |
| S 187 | 29.9 | 90.1 | 33 | S 187 | 31.3 | 100.9 | 31 |
| L 188 | 65.5 | 143.3 | 46 | R 188 | 102.8 | 204.3 | 50 |
| L 189 | 65.3 | 132.2 | 49 | L 189 | 66.4 | 133.5 | 50 |
| A 190 | 10.4 | 82.8 | 13 | A 190 | 15.7 | 90.6 | 17 |
| Y 191 | 85.8 | 171.3 | 50 | F 191 | 81.7 | 159.9 | 51 |
| D 192 | 39.1 | 128.6 | 30 | H 192 | 44.6 | 161.9 | 28 |
| Y 193 | 98.5 | 210.1 | 47 | H 193 | 83.8 | 192.5 | 44 |

Table S1: The contact areas of Nef residues in the β-catenin binding motif upon docking to ­ß-catenin. This table compares the docking HIV2-Nef DSLLAYDY and HIV1- Nef RFDSRLAFHH peptides. The table lists the contact area, exposed area and the percentage of contact area compared to exposed (Contact Area/Exposed Area) for each residue. Key residues in the motif, D186 and F191, are colored in red.
